# Screening for the protective effect target of deproteinized extract of calf blood and its mechanisms in mice with CCl_4_-induced acute liver injury

**DOI:** 10.1371/journal.pone.0180899

**Published:** 2017-07-10

**Authors:** Guangyu Xu, Xiao Han, Guangxin Yuan, Liping An, Peige Du

**Affiliations:** College of Pharmacy, Beihua University, Jilin, Jilin, China; Texas A&M University, UNITED STATES

## Abstract

Liver injury is a common pathological basis of various liver diseases, and long-term liver injury is often an important initiation factor leading to liver fibrosis and even liver cirrhosis and hepatocellular carcinoma (HCC). It has been reported that deproteinized extract of calf blood (DECB) can inhibit the replication of hepatitis B virus and confers a protective effect on the liver after traumatic liver injury. However, few studies on the regulatory factors and mechanisms of DECB have been reported. In this current study, an acute mouse liver injury model was established with carbon tetrachloride (CCl_4_). The differentially expressed genes and related cell signal transduction pathways were screened using mRNA expression microarray. STEM software V1.3.6 was used for clustering gene functions, and the DAVID and KEGG databases were applied for the analysis. A total of 1355 differentially expressed genes were selected, among which nine were validated by RT-qPCR. The results showed that the Fas, IL1b, Pik3r1, Pik3r5, Traf2, Traf2, Csf2rb2, Map3k14, Pik3cd and Ppp3cc genes were involved in the regulation of DECB in an acute mouse liver injury model. Targets of the protective effects of DECB and its related mechanisms were found in mice with acute liver injury induced by carbon tetrachloride, which may provide an important theoretical basis for further DECB research.

## 1. Introduction

Liver injury is defined as acute liver dysfunction caused by viral infection, liver toxicity, toxic substances or hepatic ischemia reperfusion, and the common pathological basis of various liver diseases is generally characterized by pathological characteristics such as liver cell degeneration, necrosis, and apoptosis [1]. Long-term liver injury can often lead to liver fibrosis and is an important initiating factor in the occurrence of liver cirrhosis and hepatocellular carcinoma (HCC) [2]. Therefore, the prevention and treatment of liver injury is a major step in the clinical treatment of liver diseases, and controlling liver injury occurrence and development hold great clinical significance for the treatment of liver diseases [3].

Main components of deproteinized extract of calf blood (DECB) include active polypeptides (molecular weight less than 5000D), nucleotides, oligosaccharides, lipid molecules, and other low-molecular weight organic matters. Inorganic substances, such as trace elements necessary for the human body and electrolytes (K^+^, Na^+^, Ca^2+^, Mg^2+^, Fe^2+^, Zn^2+^, and Cl^-^), account for 70% of DECB, and organic substances, such as oligosaccharides, nucleic acid derivatives, low molecular peptides, amino acids, lipids and sugars, and lipid metabolic intermediates, account for 30% of DECB. Small molecular peptides, which are quantitatively analyzed by the Folin-phenol reagent method, are active ingredients in DECB. Capillary zone electrophoresis and reverse phase high performance liquid chromatography (RP-HPLC) are used for fingerprint analysis of small peptides in DECB, and small peptides in DECB can be qualitatively analyzed by liquid chromatography/mass spectrometry (LC/MS). The high molecular material content and molecular weight distribution of DECB can be analyzed by gel chromatography, free amino acids and amino acids after hydrolysis of DECB can be quantitatively analyzed using RP-HPLC and precolumn derivatization, and common metal ions in DECB, including potassium, sodium, calcium, magnesium, copper, iron, zinc and lead, can be quantitatively analyzed by atomic absorption spectrophotometry [4,5]. Recent studies have shown that they can promote the growth of hepatocytes, improve the utilization of oxygen and glucose in liver cells, and enhance immune function [6]. It has been reported that DECB can inhibit replication of hepatitis B virus and that it induces a protective effect on liver function after traumatic liver injury. However, studies on DECB have mainly focused on its protection of liver injury in clinical trials or animal experiments. No in-depth study on the mechanism and related key regulatory genes has been reported, which has resulted in major limitations of the applications of DECB.

With the wide applications of CHIP and bioinformatics, the large-scale screening of drug targets and investigating of mechanisms through data mining have become a reality. In this study, an acute liver injury model induced by carbon tetrachloride (CCl_4_) was established in mice, and the differentially expressed genes and related pathways were screened using mRNA expression microarrays in DECB-treated and control groups. We sought to discover targets related to the protective effect of DECB in mice with CCl_4_-induced acute liver injury and to investigate the related mechanisms to provide a theoretical basis for further studies.

## 2. Experimental materials

### 2.1 Animals

Forty-eight-week-old male ICR mice (SPF grade), weighing 16~18 g, were purchased from Changchun City Yisi Experimental Animal Technology Co., Ltd. (license number: SCXK (Ji)-2011-0004). Animals were housed individually at a temperature of 20±1°C and a humidity of 40–70% and were subjected to a 12-h light/dark cycle with free access to food and water. The study was conducted according to the European Community Guidelines for the Use of Experimental Animals. The protocol was approved by the Ethics Committee of Beihua University.

### 2.2 Reagents

Reagents included DECB (homemade); Hugan tablets (Heilongjiang Sunflower Pharmaceutical Co., Ltd.; batch number: 201408073); an alanine aminotransferase (ALT) kit and an aspartate aminotransferase (AST) kit (Shanghai Yongle Biological Products Research Institute); glutathione (GSH), a malondialdehyde (MDA) kit and total superoxide dismutase (T-SOD) (Nanjing Jiancheng Bioengineering Institute; batch number: 20151204, 20151121 and 20151016); and a TUNEL apoptosis detection kit (Roche; batch number: 11684817910). Chemical agents for Western blot were obtained from Sigma Aldrich. All other chemical reagents were from a commercial source.

### 2.3 Instruments

Instruments included a SPX-250B-Z constant temperature biochemical incubator (Shanghai Boyuan Industrial Co., Ltd.), a 5430R low temperature high-speed centrifuge (Eppendorf company, USA) and an Infinite M200 microplate recorder (TECAN).

## 3. Experimental methods

### 3.1 Animal grouping and drug administration

After acclimating to the laboratory environment for 3 d, 40 male mice were randomly divided into 4 groups (n = 10 in each group), as follows: a blank control group (CON), a model group (M), a DECB group, and a positive control group (PC). Mice in the CON group were given normal saline (20 mL/kg), mice in the DECB group were given DECB (1 g/kg), mice in the PC group were given Hugan tablets (20 mL/kg), and mice in the M group (20 BW) were given normal saline (20 mL/kg) intragastrically once per day for 30 d.

### 3.2 Establishment of carbon tetrachloride-induced liver injury

Two hours after the last administration, mice in the CON group were injected with ablend oil, and those in the other three groups were injected with 10% carbon tetrachloride blend oil (10 mL/kg).

### 3.3 Determination of biochemical indexes and its methods

Twelve hours after the last administration, mice were deprived of food but not drinking water for 24 h. Mice were anesthetized with 1% pentobarbital sodium (80 mg/kg), and blood samples were taken from their peripheral blood and centrifuged to obtain the serum. Alanine transferase (ALT) and aspartic acid transferase (AST) activities in the serum were detected according to the kit instructions. The liver tissues of the mice were washed with cold normal saline and then prepared into 10% liver homogenates; the homogenates were centrifuged to obtain the sera. T-SOD activity, GSH and MDA contents were determined according to the kit instructions.

### 3.4 HE staining of liver tissues

The liver tissues of the mice were fixed with 10% neutral formalin, embedded with paraffin, sliced, stained with hematoxylin-eosin (HE) and observed under a light microscope.

### 3.5 Detection of liver cell apoptosis

Apoptosis of liver cells was detected by TUNEL assays. According to the kit instructions, liver cells were labeled using TUNEL, after which the nuclei of apoptotic cells with positive TUNEL staining showed a dark brown color.

### 3.6 Western blot analysis

Detection of caspase-8, caspase-3 and caspase-9 protein contents in the liver tissue by Western blot: An appropriate amount of frozen liver tissue was thawed on ice and then treated with cell lysate and centrifuged to obtain the supernatant for sodium dodecyl sulfate polyacrylamide gel electrophoresis (SDS-PAGE). For SDS-PAGE, the protein was transferred onto a nitrocellulose membrane, blocked with TBS containing 5% skim milk, supplemented with the first antibody (1:4000 diluted), incubated at 37°C for 1 h, washed 3 times with TBS containing 5% skim milk for 10 min each time, supplemented with the secondary antibody (1:000 diluted), incubated at 37°C for 1 h, and washed 3 times with TBST for 10 min each time, and the images were obtained by ECL blotting chemiluminescence. β-actin was used as the reference protein, ImageJ was used to determine the gray values of the bands, and the amount of target protein expression corresponding to that of the reference was determined by the gray comparison method for analyzing the difference in the amount of protein expression among the samples.

### 3.7 RNA extraction and quality control

Total liver RNA was extracted using Trizol reagent (Invitrogen, Gaithersburg, MD, USA), and the mRNA was purified according to the instructions of the RNeasy mini kit (Qiagen, Valencia, CA, USA). RNA samples were assessed for RNA integrity, inhibitor, and DNA contamination according to the kit instructions. The quality of mRNA was detected by agarose gel electrophoresis, and the content was determined by a UV spectrophotometer.

### 3.8 Microarray experiment

#### 3.8.1 Sample labeling and hybridization

The samples were labeled in accordance with the instructions of the Quick Amp Labeling Agilent kit, and hybridization experiment was carried out using SureHyb Agilent.

#### 3.8.2 Data acquisition and standardization

The micro-array was scanned with an Agilent DNA Microarray Scanner after washing. Agilent Feature Extraction software (v11.0.0.1) was used for acquisition of chip probe signal values. The chip standardization was conducted with Agilent GeneSpring GX v12.1 software. Points outside the 95% confidence interval were considered differentially expressed genes.

#### 3.8.3 Analysis of differentially expressed genes and their functions

Agilent Gene Spring GX v12.1 software was used to select the differentially expressed genes.

#### 3.8.4 Cluster analysis

Clustering analysis of significantly differentially expressed genes was carried out using STEM software V1.3.6 (short time-series expression miner, http://www.cs.cmu.edu/jernst/stem/). Based on the norms of the software, the gene list to be analyzed was constructed, Mouse (EBI) was selected as the functional annotation gene pool, Mus musculus (ensemble/Biomart) was used for the gene mapping database, and the operation was performed after setting the time expression module number to obtain the expression modules of significantly differential genes (known as the clustering phase) for which *P* < 0.001 was taken as the screening standard for significance.

#### 3.8.5 Analysis of the functional significance of differentially expressed genes

Based on the NCBI gene ontology database, GO annotation of these genes was conducted to obtain all GOs in which the genes were involved, the Fisher exact test and Chi square test were used to calculate the significance level and misjudgment rate of each GO, and the P values were calibrated with the misjudgment rate to screen for the significance (*P* < 0.05) of differential genes [7]. The results were artificially analyzed with the European Bioinformatics Institute (EBI) database.

#### 3.8.6 Analysis of the function and biological pathway enrichment of differential genes screened by the mRNA expression microarray using DAVID

The DAVID database (http://david.abcc.ncifcrf.gov) was opened, and 1355 genes were presented as the gene set for further analysis. In addition, the corresponding gene identifier (the gene identifier corresponding to the gene name was OFFICIAL_GENE_SYMBOL) was selected; the whole mouse genome mice was marked as background genes, and the "functional annotation tool" was selected as the analytic tool, through which the results of GO and biological pathway enrichment analysis of differentially expressed genes were obtained [8].

#### 3.8.7 KEGG analysis, data sources and pretreatment

All pathways used in this study were obtained from the KEGG database [9]. Apoptosis-relevant KEGG pathways were obtained from http://www.kegg.jp/kegg-bin/show_pathway?map04210 in April 2016.

### 3.9 Diseases and biofunction analysis

Ingenuity Pathway Analysis (IPA) analysis was performed through the Ingenuity^®^ Knowledge Base database and subjected to the Fisher exact test. Through IPA analysis, we obtained significant diseases and biofunctions and experimental data corresponding to biological trends. Through this analysis, we identified the gene group with the specific biological function, predicted the activation and inhibition of biological functions, and provided detailed data for the direct understanding of the intrinsic cause of a specific biological phenotype.

### 3.10 Validation by real-time quantitative PCR

The differentially expressed genes relevant to the regulation of DECB on mice immune functions were validated by real-time quantitative PCR. The operation of RT-qPCR was strictly in accordance with the Real-time PCR Master Mix (SYBR Green, TOYOBO Company) manual, and the instrument used was a Roche LightCycler 1.5. The primers are shown in Table 1.

**Table 1:** Primer pairs for real-time PCR.

Gene name	Sequences of two way primers	Annealing temperature (°C)	Product length (bp)
GAPDH	F:5’CACTGAGCAAGAGAGGCCCTAT3’ R:5’GCAGCGAACTTTATTGATGGTATT3’	60	144
Fas	F:5’ ATCTGGGCTGTCCTGCCTCTG3’ R:5’ TCAGTTTCACGAACCCGCCTC3’	60	113
Traf2	F:5’ CAGAAAGCGTCAGGAAGCCGTAG3’ R:5’ CAGTGCCGTCGCCATTCAAGT 3’	60	119
Il1b	F:5’ CTTCAGGCAGGCAGTATCACTC3’ R:5’ GCAGTTGTCTAATGGGAACGTC3’	60	194
Pik3r1	F:5’ TCTACCCAGTGTCCAAATACCAG3’ R:5’ TAAATGCTTCGATAGCCGTTC3’	60	189
Pik3r5	F:5’ ATCGGATTTCAGGGAAGGTGG3’ R:5’ GGAGTGGAGATGCTTGGCTCA3’	60	174
Ppp3cc	F:5' TTCCTCTTGCTGCCCTCTTAA3’ R:5’ TTCTCGCTGCCGTAGTCCTCT3’	60	175
Pik3cd	F:5' CATCAGTGGCTCTGCGGTTTG3’ R:5’ ATCTCCTTGGTTTGGGGCTTG 3’	60	169
Csf2rb2	F:5' GGACATAGAGTTTGAGGTGGC3’ R:5’ AGCATAGATGCTGTTGGGTAG3’	60	127
Map3k14	F:5' AAGACCGAGCCCTTTACTACC3’ R:5’TCAGCTTTGACATCGCCATGC3’	60	91

### 3.11 Data processing

SPSS 16 software was used for data analysis. Single-factor analysis of variance and q tests on the measurement data were conducted with the ANOVA program, the least significant difference (LSD) test was used for pairwise comparisons. The graded data were analyzed with Ridit, and *P* < 0.05 was considered statistically significant [10].

## 4. Results

### 4.1 Effects of DECB on liver functions of mice with CCl_4_-induced hepatic injury

Compared with those in the blank control group, serum ALT and AST activities of mice in the model group were significantly elevated (*P* < 0. 05). Compared with those in the model group, serum ALT and AST activities of mice in the DECB and positive control groups were significantly reduced (*P* < 0. 05), as shown in Table 2.

**Table 2:** Effects of DECB serum ALT and AST activities in mice with CCl_4_-induced liver injury.

Group	AST (IU/L)	ALT (IU/L)
CON	61.38±6.42	25.19±5.96
M	97.73±9.33^#^	56.46±11.30^#^
DECB	65.34±15.39*	33.01±5.86*
PC	63.70±7.48*	35.04±3.99*

*P<0.05 *vs*. model group,

^#^*P*<0.05 *vs*. blank control group.

CON, blank control group; M, model group; DECB, deproteinized extract of calf blood group; PC, positive control group.

### 4.2 Effects of DECB on GSH, MDA and T-SOD in the liver tissues of mice with CCl_4_-induced liver injury

GSH and T-SOD activities and MDA levels of liver tissues in the model group significantly differed from those in the blank control group (*P* < 0.01). Compared with those in the model group, GSH activity was significantly increased (*P* < 0.05), MDA level was significantly reduced (*P* < 0.05), and T-SOD level was significantly reduced (*P* < 0.05) in the DECB group and the positive control group (Table 3).

**Table 3:** Effects of DECB on GSH, MDA and T-SOD in liver tissues of mice with CCl_4_-induced liver injury.

Group	GSH /mg·g^-1^	MDA /nmol·mg^-1^	T-SOD U/mg prot
CON	1.32±0.27	0.60±0.08	278.08±30.62
M	4.18±1.99^#^	1.24±0.64^#^	164.41±63.57^#^
DECB	1.21±0.45*	0.56±0.22*	247.41±37.89*
PC	1.01±0.13*	0.45±0.14*	254.6±43.12*

*P<0.05 vs. model group,

^#^*P*<0.05 *vs*. blank control group.

CON, blank control group; M, model group; DECB, deproteinized extract of calf blood group; PC, positive control group.

### 4.3 Effects of DECB on pathological changes in the liver tissue of mice with CCl_4_-induced liver injury

**Fig 1:**
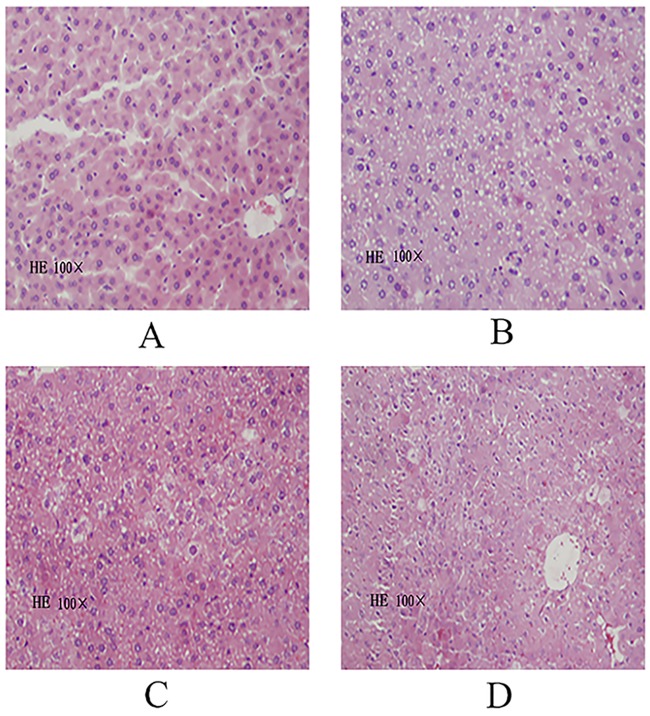
HE staining of the liver cells of mice (HE100×). (A: blank control group; B: model group; C: DECB group; D: positive control group).

The pathological changes of liver tissue were observed under a light microscopy. The results showed that liver cells were orderly arranged with clear nucleoli and contained abundant cytoplasm with a red color and round nuclei. The nuclear chromatin appeared blue, and the size of the liver sinus was normal. No abnormal pathological changes in liver tissues in the blank control group were observed (Fig 1A). In the model group, liver cells were markedly injured, presenting obvious liver cell swelling, with irregular nucleus size, varying degrees of shrinkage, hepatic cord disorder, loose cytoplasm, varying degrees of liver cell degeneration, fatty degeneration of a large number of liver cells, reticulate structure with pale red loose cytoplasm, lipid droplet vacuoles of different sizes in the cytoplasm, and lymphocytic infiltration, but no obvious liver fibrosis (Fig 1B). In the DECB and positive control groups, sporadic lipid droplets and cells with hydropic degeneration were observed in liver tissues, but to a lesser degree than in the model group, and liver cells were mildly swollen (Fig 1C and 1D).

### 4.4 Effect of DECB on liver cell apoptosis

The apoptosis of liver cells was detected by TUNEL staining, in which the nuclei of apoptotic liver cells were brownish yellow and those of non-apoptotic liver cells were blue.

**Fig 2:**
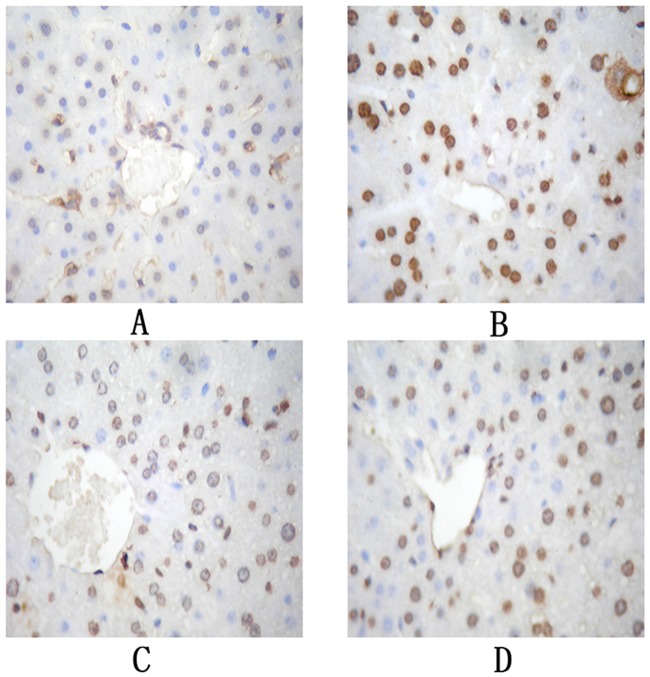
TUNEL staining of the liver of mice cells (×400). (A: blank control group; B: model group; C: DECB group; D: positive control group).

Compared with those in the blank control group, the number of cells with positive TUNEL staining in the liver tissue in the model group and the apoptosis index were significantly increased (*P* < 0.01). The cells in the DECB group were significantly reduced and the apoptosis index was significantly decreased (*P* < 0.01) compared with those in the model group, as shown in Fig 2 and Table 4.

**Table 4:** Effect of DECB on the hepatocyte apoptosis index in mice with CCl4-induced liver injury (*n* = 10, mean±SD).

Group	Apoptosis index (%)
CON	0.63±0.38
M	6.09±0.78^#^
DECB	2.89±0.55*
PC	2.65±0.46*

*P<0.05 vs. model group,

^#^*P*<0.05 *vs*. blank control group.

CON, blank control group; M, model group; DECB, deproteinized extract of calf blood group; PC, positive control group.

### 4.5 Western blot analysis

**Fig 3:**
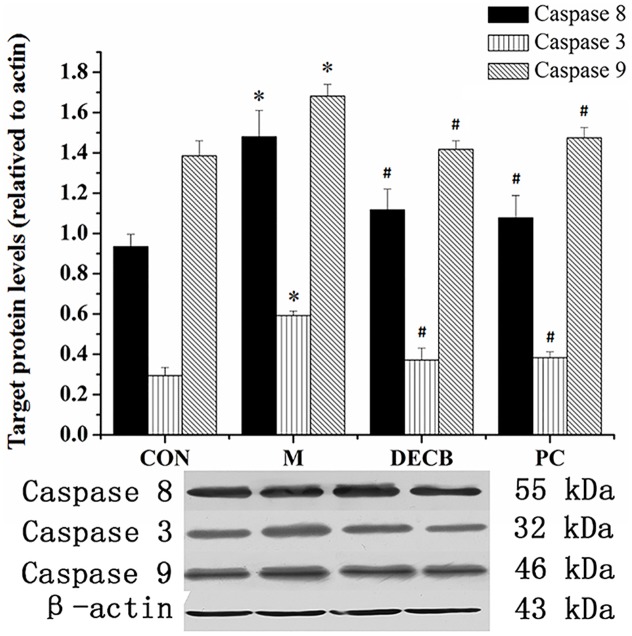
Weston blot validation of caspase-8, caspase-3 and caspase-9. *P<0.05 vs. blank control group, ^#^*P*<0. 05 vs. model group. (CON, blank control group; M, model group; DECB, deproteinized extract of calf blood group; PC, positive control group).

There was only a small amount of caspase-3, caspase-8 and caspase-9 expressed in the normal liver tissues, and the expression of caspase-3, caspase-8 and caspase-9 increased (*P*<0.05) in the model group but decreased in the DECB and PC groups (*P*<0.05), as shown in Fig 3.

### 4.6 Screening of differentially expressed genes by mRNA expression microarray

**Fig 4:**
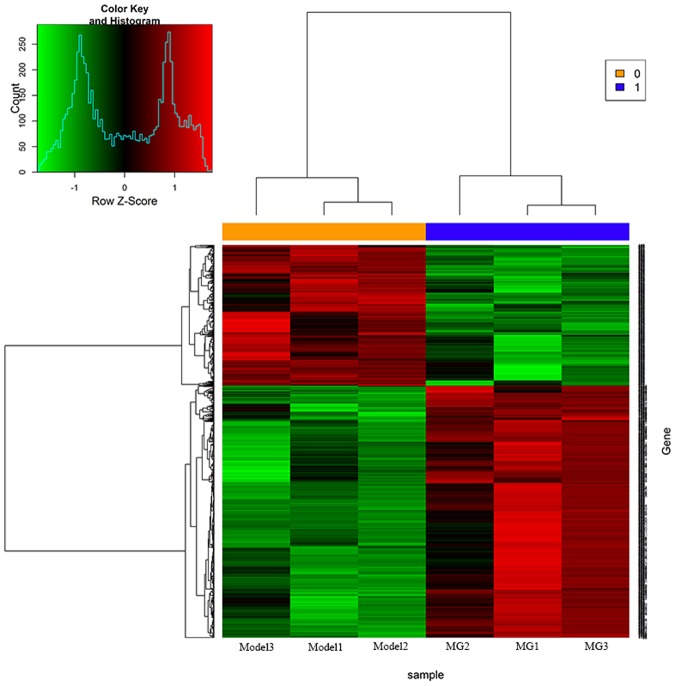
Results of mRNA expression profile microarray analysis. (M, model group; DECB, deproteinized extract of calf blood group).

The results of differentially expressed genes screened by mRNA expression microarrays showed that there were 1355 differentially expressed genes in the DECB group compared with the model group, among which the expression of 507 (30.49%) genes was significantly up-regulated, and the expression of 848 (69.51%) genes was significantly down-regulated (see Fig 4).

In this experiment, the liver injury in the mice was induced by CCl_4_. It is well known that liver injury is closely related to apoptosis of liver cells and that the promotion of liver cell apoptosis can cause liver injury. On the contrary, the inhibition of liver cell apoptosis may prevent liver injury and thus protect the liver. As shown in Fig 2, the results obtained by mRNA expression microarray analysis in the DECB group and the model group revealed an obvious distinction, indicating that DECB could protect the liver by inhibiting apoptosis-related genes (69.51% of gene expression was significantly down-regulated).

### 4.7 Clustering analysis of mRNA expression profile microarray data

Clustering analysis with STEM V1.3.6 software was performed for the 1355 differentially expressed genes, and the results revealed nine differentially expressed genes in the apoptotic pathway, among which the expression of 2 genes was up-regulated and the expression of the other 7 genes was down-regulated (Table 5).

**Table 5:** Differential expression of genes in the apoptotic pathway based on the mRNA microarray.

EntrezGeneID	GeneSymbol	Regulation	P-value	FDR	Description
14102	Fas	down	0.000657175	0.083560716	Fas (TNF receptor superfamily member 6)
18708	Pik3r1	down	0.00125786	0.098754118	phosphatidylinositol 3-kinase, regulatory subunit, polypeptide 1
22030	Traf2	up	0.00494606	0.143934526	TNF receptor-associated factor 2
320207	Pik3r5	down	0.005657697	0.150032002	phosphoinositide-3-kinase, regulatory subunit 5
19057	Ppp3cc	up	0.010725845	0.180866379	product:protein phosphatase 3, catalytic subunit, gamma isoform, full insert sequence
18707	Pik3cd	down	0.018633533	0.215061721	phosphatidylinositol 3-kinase catalytic delta polypeptide
16176	Il1b	down	0.0209536	0.223322644	interleukin 1 beta
12984	Csf2rb2	down	0.04246625	0.278562932	colony stimulating factor 2 receptor, beta 2, low-affinity
53859	Map3k14	down	0.046223273	0.285394112	mitogen-activated protein kinase kinase kinase 14

### 4.8 Diseases and biofunction analysis

We used an IPA system to analyze the mRNA expression microarray data and found that the most relevant diseases mainly included immunological disease, endocrine system disorders, gastrointestinal disease, organismal injury, metabolic diseases and infectious diseases. The most relevant biological function was apoptosis of stem cell clonogens (Tables 6 and 7).

**Table 6:** Related diseases of IPA analysis.

Diseases	Functions Annotation	p-Value
Immunological Disease	systemic autoimmune syndrome	4.83E-65
Endocrine System Disorders, Gastrointestinal Disease, Immunological Disease, Metabolic Disease, Organismal Injury and Abnormalities	insulin-dependent diabetes mellitus	2.37E-57
Endocrine System Disorders, Gastrointestinal Disease, Metabolic Disease, Organismal Injury and Abnormalities	diabetes mellitus	7.29E-48
Hematological System Development and Function, Tissue Morphology	quantity of leukocytes	4.59E-44
Cellular Movement, Immune Cell Trafficking	leukocyte migration	1.18E-40
Hematological System Development and Function, Tissue Morphology	quantity of blood cells	4.08E-40
Metabolic Disease	glucose metabolism disorder	1.36E-39
Cellular Movement, Hematological System Development and Function, Immune Cell Trafficking	cell movement of leukocytes	4.74E-37
Infectious Diseases	infection of mammalia	1.46E-34

**Table 7:** Related biofunctions of IPA.

Bio function	Functions Annotation	p-Value
Cell Death and Survival	apoptosis of stem cell clonogens	0.000633
Gene Expression	inactivation of mouse X chromosome	0.000633
Cell Morphology, Cellular Compromise, DNA Replication, Recombination, and Repair	mutagenesis of lung cells	0.000633
Cell Morphology, Cellular Compromise, DNA Replication, Recombination, and Repair	mutagenesis of splenocytes	0.000633
Cellular Development, Cellular Growth and Proliferation, Hematological System Development and Function, Inflammatory Response, Lymphoid Tissue Structure and Development	proliferation of mononuclear phagocyte progenitors	0.000633
Cell Death and Survival	survival of stem cell clonogens	0.000633
Behavior, Nervous System Development and Function	circadian rhythm	0.000889
Lipid Metabolism, Small Molecule Biochemistry	synthesis of diacylglycerol	0.00123
Embryonic Development, Organismal Development, Tissue Development	development of neuroectoderm	0.00125

### 4.9 Real-time quantitative PCR validation

**Fig 5:**
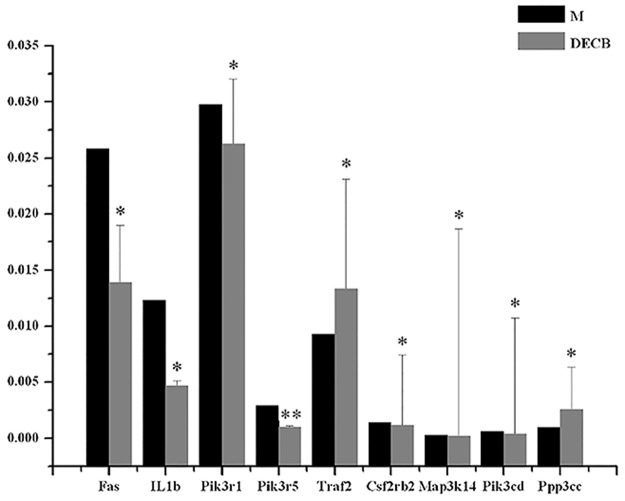
Real-time quantitative PCR validation of the Fas, IL1b, Pik3r1, Pik3r5, and Traf2 genes. *P<0.05, **P<0.01, DECB group *vs*. model group (M, model group; DECB, deproteinized extract of calf blood group).

The Fas, IL1b, Pik3r1, Pik3r5, and Traf2 genes were selected for real-time quantitative PCR (Table 8 and Fig 5). The results showed that the Fas, IL1b, PIK3R1, and Pik3r5 genes played a significant role in the CCl_4_-induced mice liver injury model (p-values = 0.00510, 0.00048, 0.00572, and 0.00006, respectively), their expressions were down-regulated, and DECB inhibited the expression of these four genes (the ratios of DECB group/model group for these genes were 0.54, 0.38, 0.88, and 0.35, respectively). Expression of the Traf2 gene was up-regulated in the model group, which was highly significant (*P* = 0.00984), and DECB could promote the expression of this gene (the ratio of the DECB group/model group was 1.44).

**Table 8:** Results of real-time quantitative PCR validation.

Sample No.	Fas/GAPDH	IL1b/GAPDH	Pik3r1/GAPDH	Pik3r5/GAPDH	Traf2/GAPDH	Csf2rb2/GAPDH	Map3k14/GAPDH	Pik3cd/GAPDH	Ppp3cc/GAPDH
AVE M	2.58E-02	1.23E-02	2.98E-02	2.94E-03	9.26E-03	1.42E-03	2.51E-04	6.01E-04	9.71E-04
AVE DECB	1.39E-02	4.65E-03	2.63E-02	1.05E-03	1.33E-02	1.15E-03	1.74E-04	3.77E-04	2.57E-03
DECB/M	0.54	0.38	0.88	0.35	1.44	0.81	0.69	0.63	2.65

## 5. Discussion

CCl_4_-induced liver injury model is a classical model that is commonly used to study the liver, liver drug efficacy and mechanisms of action [11]. The currently accepted mechanism of CCl_4_-induced liver injury is that CCl_4_ can generate CCL_3_ and CCl_3_O_2_ radicals through activation of hepatic microsomal cytochrome P450. These free radicals can covalently bind to macro-molecules in liver cells and attack unsaturated lipids under the cytoplasmic membrane to induce lipid peroxidation (LPO). LPO and its degradation products can also cause damage to the stability and integrity of a variety of biological membranes and increase their permeability, resulting in the outflow of enzymes in the cytoplasm into the blood, such as ALT and AST [12,13]. Therefore, the determination of ALT and AST activities in the serum is an important index for evaluating liver injury. In the current experiment, the activity of serum ALT and AST in the DECB group was significantly lower than that in the model group, suggesting that DECB may exert a protective effect on CCl_4_-induced acute liver injury. The metabolic end-product MDA of lipid peroxides can reflect the degree of oxidation reaction and induce cell damage in the body [14,15]. SOD is the main defensive antioxidant enzyme in cells, which can clear free radicals, mitigate the chain reaction of lipid peroxidation, and protect the structural stability and functional integrity of membranes. It is believed that MDA content and SOD activity can indirectly reflect the level of oxygen free radicals, and the GSH level can reflect the body's antioxidant capacity [16]. The results showed that SOD activity and GSH level were significantly increased, while MDA content was significantly decreased in the DECB group, indicating that DECB can enhance the antioxidant capacity of the liver and reduce the formation of lipid peroxides. DECB extracted and purified from calf serum by ultrafiltration and concentration technologies mainly consists of small molecular substances, such as inorganic ions (potassium, sodium chloride, etc.), small peptides, amino acids, and sugars. Polypeptide concentrations and the contents of DECB products from various manufacturers differ. Higher contents of polypeptides confer stronger in vivo antioxidant ability, suggesting that the effective antioxidant components of DECB could be the polypeptides [4].

We found that its most important biological function was on stem cell apoptosis through the IPA analysis of mRNA expression microarray. As is well known, apoptosis can lead to a variety of diseases, including cancer, autoimmune diseases, and cardiovascular disease. In addition, we also found through IPA analysis that related diseases included immune system diseases, endocrine system diseases, blood system diseases, metabolic diseases and infectious diseases. Therefore, our focus was on cell apoptosis, confirming that the effect of DECB could be related to the apoptosis of cells.

Liver cells undergo death via the two primary mechanisms of necrosis and apoptosis. Apoptosis is the first response of liver cells to various injury factors, and necrosis tends to occur following apoptosis. The apoptosis process plays a crucial role in the formation of necrosis among liver cells [17,18]. Therefore, studies on the mechanism of apoptosis are important to further reveal the nature of CCl_4_-induced acute liver injury and liver failure. The results revealed that apoptosis of hepatocytes was obvious, and the apoptosis index was increased in CCl_4_-induced acute liver injury. Oxidative stress induced by reactive oxygen species (ROS) is an important link in causing apoptosis. SOD is one of the main free radical scavenging enzymes in the body. The decrease of effective hepatic blood flow causes the decrease of SOD, an endogenous scavenger of oxygen free radicals, and meanwhile the synthesis of SOD is also decreased, leading to the deficiency of oxygen free radical scavenging, which in turn inhibits the activity of SOD further. The decreased SOD can trigger the catalytic cracking of lipid peroxides in the presence of metal ions to generate MDA, and MDA via a cytotoxic effect can bind to proteins through intramolecular and intermolecular cross-linking molecules to induce apoptosis [16] The apoptosis index significantly reduced after the administration of DECB, suggesting that DECB could inhibit the apoptosis of liver cells, which may be achieved through regulating the expression of apoptosis-related genes. The activation of caspases occurs repeatedly in apoptosis mediated by oxidative stress, which has an important influence on the apoptosis process. Caspases are the ultimate factors by which apoptosis is promoted, play key roles in the process of mediating apoptosis, and are the rendezvous point of many apoptotic pathways. Thus, caspases are ultimate factor responsible for executing apoptosis. In addition, another mechanism through which caspases function is self-activation through their mutual activation, and once they initiate the process of apoptosis, the cascade effect is initiated [19]. In this experiment, caspase-3, caspase-8 and caspase-9 expression increased in liver tissues in the model group, indicating that the activation of caspase-8 and caspase-9 can activate caspase-3 to initiate a caspase cascade reaction to induce apoptosis of liver cells. The RT-qPCR results in this experiment showed increased Fas expression, suggesting that Fas could directly interact with the endogenous Fas-associated death domain to generate an apoptosis complex to bind to pro-caspase-8, activating pro-caspase-8 to become a caspase with activity [20,21]. Activated caspase-8 can further activate caspase-3 in the downstream cascade, eventually leading to the apoptosis of cells. Caspase-9, one of the most important apoptosis-initiating factors, is located in the cascade upstream. It can complete its self-activation in the presence of other proteins and activate downstream caspase factors, such as caspase-3, and then induce a cascade amplification effect, thereby leading to cell apoptosis. Caspase-3 is an important apoptosis factor and a key enzyme of apoptosis. A cascade reaction will occur downstream once caspase-3 is activated, and thereafter, apoptosis is inevitable [22]. In this study, the expression of caspase-3, caspase-8 and caspase-9 decreased in DBCE group, indicating that DBCE can alleviate apoptosis by inhibiting the caspase cascade, thus inhibiting apoptosis. The Fas, IL1b, PIK3R1 and Traf2 genes all are closely related to apoptosis. Studies have found that both Fas and Traf2 are involved in the TNF signaling pathway: Fas, IL1b, and PIK3R1 can promote cell apoptosis, and Traf2 can inhibit cell apoptosis. The results of screening for differentially expressed genes revealed a down-regulation of Fas, IL1b and Pik3r1 and an up-regulation of Traf2 in the model group and the DECB group. Related studies have shown that Fas, Pik3r1 and TRAF2 are all involved in cell signaling pathways, and IL1b is involved in the regulation of inflammatory responses, while studies of Pik3r5 are still currently lacking.

Tumor necrosis factor receptor-associated factors (TRAFs) are important components in the signal transduction of the TNF receptor super family and the IL-1R/TLR super family [23]. TRAF2 is one of the most extensively expressed TRAF family members and plays an important role in TNF signal transduction of almost all branching pathways as an adaptor protein and regulatory factor [24]. TRAF2 can interact with anti-apoptotic factors to inhibit cell apoptosis [25,26]. The results obtained by the mRNA expression microarray assay showed that expression of Traf2 was significantly up-regulated (*P*<0.01), and DECB could promote the expression of Traf2 and then inhibit the apoptosis of liver cells.

As one of the important signal transduction pathways in cells, the PI3K/AKTA/mTOR pathway plays an important biological role in the process of growth, survival, proliferation, apoptosis and angiogenesis [27]. PI3K family members are important kinases of phosphatidylinositol (PI) and important intracellular signaling molecules, which are involved in the regulation of cell proliferation, apoptosis and differentiation [28]. Pik3r1 and Pik3r5 are PI3K family members [29]. The PIK3R1 (phosphoinositide-3-kinase, regulatory subunit 1 and p85alpha) gene encodes the 85kD regulatory subunit p85α of phosphatidylinositol 3-kinase (PI3K). p85 is the regulatory subunit primarily expressed in the PI3K family, and all related signaling molecules are required to bind to the subunit p85 to activate PI3K. Then, through a variety of signaling pathways, cell growth, differentiation, adhesion and cell cycle processes are affected to promote the proliferation, invasion and metastasis of tumor cells and to inhibit apoptosis of tumor cells. The up-regulation of pik3cd is common in malignant tumors [30,31]. The down-regulation of pik3cd in the mRNA chip experiment indicated that it was inhibited by EDCB in the mouse model. Few studies on Pik3r5 have been reported, and none have shown that it is significantly correlated with apoptosis [32]. In the current study, quantitative real-time PCR revealed a p value of 0.00006 for Pik3r5, and its experimental results were more significant than those of the other four genes, indicating that the Pik3r5 gene is closely related to the protective effects of DECB on liver injury and can be investigated as a potential target of DECB.

The IL1b gene encodes IL1β. IL1β is a pro-inflammatory cytokine and belongs to the IL1 family, which mainly includes α and β monomers. Activities of the IL1 family are mainly expressed through IL1β and are involved in the regulation of acute and chronic cell inflammatory reactions [33,34]. IL1β is primarily produced by blood monocytes and tissue macrophages and can induce the expression of genes involved in a variety of inflammatory reactions. It can induce the free radical effect, followed by a series of inflammatory reactions, such as the induction of tumor necrosis factor-α and the oxygen effect, resulting in activation of the nuclear transcription factor kB signal transduction pathway [35]. Studies have indicated that the up-regulation of IL1β expression is involved in breast cancer, colon cancer, melanoma and lung cancer and promotes cell apoptosis [36]. The results of mRNA expression microarray analyses showed that the down-regulated expression of IL1β was not significant (*P* = 0.0209536), indicating that DECB could inhibit the expression of IL1β, although the difference was not significant. Real-time quantitative PCR showed that the expression of IL1β was also down-regulated (*P* = 0.00048), indicating that IL1β could be used as a potential drug target, although it has poor stability (Table 5).

The Map3k14 gene is closely related to NF-kappa B-inducing kinase (NIK) and has been correlated with certain cancers [37]. The Csf2rb2 gene has been rarely reported until recently. RT-qPCR revealed that it had high significance (*P* = 0.00626585), and therefore, it could be regarded as a potential drug target. Both the Map3k14 gene and the Csf2rb2 gene were down-regulated in the mRNA chip experiment, indicating that both were inhibited by DECB.

Excessive apoptosis can lead to neuronal degeneration, and neuronal degeneration can lead to schizophrenia. The ppp3cc gene is a gene linked to susceptibility to schizophrenia [38], and it has been reported that the ppp3cc gene is also associated with prostate cancer [39]. Therefore, ppp3cc may also be associated with cell apoptosis. RT-qPCR revealed that the ppp3cc gene was highly significant (*P* = 0.003736), which should be further evaluated in future research studies of apoptosis.

## 6. Conclusion

The Fas, IL1b, PIK3R1, Pik3r5, Traf2, Csf2rb2, Map3k14, Pik3cd and Ppp3cc genes were closely associated with cell apoptosis. Fas, Pik3r, Traf2, Ppp3cc and Pik3cd may be important factors of apoptosis-related signaling pathways, and IL1β could regulate and control cellular inflammatory responses and promote cell apoptosis. Although a few studies on Csf2rb2, Map3k14 and Pik3r5 have been recently published, the highly significant *P* value revealed by the RT-qPCR results indicates that Pik3r5 may be the key factor of apoptosis.
